# A Novel Determination of the Foreshock ULF Boundary: Statistical Approach

**DOI:** 10.1029/2024JA033195

**Published:** 2024-11-29

**Authors:** A. Salohub, J. Šafránková, Z. Němeček, G. Pi

**Affiliations:** ^1^ Faculty of Mathematics and Physics Charles University Prague Czech Republic

**Keywords:** solar wind, foreshock, bow shock, ULF waves, ULF wave foreshock boundary

## Abstract

The location and spatial extent of the region populated by the foreshock waves depend on the IMF orientation. We performed a systematic statistical study of wave activity in the frequency range of 0.03−0.15 Hz observed during an initial phase of the THEMIS mission. Wave activity is quantified by standard deviations of the IMF magnitude and its components over 10‐min intervals. We apply the foreshock coordinate system defined as the angle between the bow shock normal and upstream magnetic field vectors and the distance from the spacecraft to bow shock along the magnetic field line. We have found that the Ultra‐low Frequency (ULF) foreshock boundary (a) is well defined in these coordinates, (b) it tends to shift outward with an increasing solar wind bulk speed, and (c) with an increasing Mach number. However, the change of the fluctuation level in the foreshock is not uniform because the increasing solar wind bulk speed enhances the fluctuation level mainly in a close proximity of the bow shock whereas the increasing Mach number leads to an intensification of fluctuation levels at the foreshock boundary.

## Key Points

- Ultra‐low frequency (ULF) foreshock boundary tends to shift outward with an increasing solar wind bulk speed
- ULF foreshock boundary moves outward with an increasing Mach number
- Solar wind speed and foreshock fluctuation amplitude are positively correlated in a close proximity of the bow shock

## 1 Introduction

Ultra‐low‐frequency (ULF) waves originating in the foreshock (a region formed upstream the bow shock when the interplanetary magnetic field has a significant radial component) play an important role in modulating the shape of the bow shock front and affect particle reflection at the shock. It is believed that they are the main source of magnetospheric Pc3 waves in the range of tens of mHz, suggesting wave transmission (Takahashi et al., 1984). The compressional Pc3 waves observed in the dayside magnetosphere modulate energetic particle precipitation into the upper atmosphere (Motoba et al., 2019). The enhanced ULF wave activity is typically associated with geomagnetic storms (Sandhu et al., 2021). Nevertheless, after decades of intensive research, it is still unclear how foreshock waves traverse through the bow shock and magnetosheath and under what conditions (Turc et al., 2023; Zhang & Liu, 2023). Combining global numerical simulations and spacecraft observations, Turc et al. (2023) demonstrate that the interaction of ULF foreshock waves with the shock generates earthward‐propagating, fast‐mode waves, which reach the magnetosphere. Other alternative transmission mechanisms, as mode conversion and shock reformation may play a particular role in the subsolar magnetosheath because only magnetosonic waves are able to reach the magnetopause. These findings give an important insight into the interaction of foreshock waves with collisionless shocks in general, and demonstrate their impact on the downstream medium. Although it seems that the importance of the foreshock waves in the solar wind–magnetosphere interaction is crucial, they occupy only a very limited region bounded by the bow shock on the downstream side and by the so‐called ULF foreshock boundary on the other side. The location of this outer boundary is discussed from the beginning of space investigations but there are still non‐conclusive results and this is the principal motivation of the present study.

The foreshock is a complex environment permeated by ions reflected from and accelerated at the bow shock (Meziane et al., 2004). These ions move along the upstream interplanetary magnetic field (IMF) lines but their motion is affected by the solar wind motional electric field and thus the reflected ions are subject of E×B drift directed toward the bow shock under a typical IMF orientation (Andres et al., 2015). The backstreaming ions interact with the incoming solar wind (Burgess et al., 2012; Eastwood et al., 2005; Fuselier et al., 1986; Greenstadt, 1976; Hoppe et al., 1981; Wilson III, 2016) and excite various types of waves with large amplitudes (Hoppe & Russell, 1983; Thomsen, 1985).

In accord with generation mechanisms, the wave frequencies lie in the ULF range and are usually classified as fast magnetosonic (Gary, 1991) or Alfvén/Ion cyclotron (Blanco‐Cano & Schwartz, 1997) waves. To give an example, they can be observed as quasi‐monochromatic wave packets with a frequency of 0.03 Hz (Fairfield, 1969) or so called 1 Hz waves (Fairfield, 1974; Hoppe & Russell, 1981; Wilson III, 2016). The waves generally propagate upstream in the plasma rest frame (Narita et al., 2004) but they are advected antisunward in the bow shock frame. However, clearly defined wave modes are observed only exceptionally and close to the foreshock boundaries due to nonlinear interactions between different wave modes, generating a broad frequency spectrum of intermittent fluctuations (Narita et al., 2006). The authors demonstrate that the fluctuation power and intermittency are significantly greater in directions perpendicular to the mean magnetic field and it suggests that the dominant source of the observed fluctuations is the interaction of Alfvén waves. These mechanisms contribute to the creation of a well‐defined boundary upstream of the bow shock, dividing the space occupied by the undisturbed solar wind from the region where these processes occur. Depending on the subject of a particular study, this boundary is referred to as the foreshock ULF boundary or the ion foreshock wave boundary (Eastwood et al., 2005).

Inspecting the magnetic field or plasma parameters time series, the ULF boundary can be identified as a rapid increase of the fluctuation power. Based on this feature, numerous studies, including simulation ones, tried to find its location and shape at the Earth (Blanco‐Cano et al., 2009; Kajdič et al., 2021; Omidi et al., 2009, 2013), near Venus (Shan et al., 2018), around Saturn (Andres et al., 2013), Mercury (Romanelli et al., 2020) and Mars (Shan et al., 2020).

It should be noted that, although the ion foreshock boundary is relatively well defined for the interplanetary magnetic field (IMF) orientation along the Parker spiral, energetic particles of the bow shock origin together with the low‐frequency waves can be observed in larger distances, including the lunar orbit (Jurac & Richardson, 2001; Salohub et al., 2022) or even as far as 80 $R_{E}$ upstream (Berdichevsky et al., 1999; Meziane et al., 2001), especially if the IMF points radially.

One of the first attempts to determine the boundary between the pristine solar wind and wave activity region was performed by Greenstadt and Baum (1986) using the ISEE magnetometer data. The authors demonstrate the existence of a boundary for the ULF compressional wave region. The ULF foreshock boundary was identified as the spacecraft passes from a region where only weak IMF fluctuations were detected into the region where strong ULF waves are observed. The boundary is characterized by a sudden variation of all three IMF components on a small time scale but the average orientation holds steady. This feature enables an introduction of the Solar Foreshock Coordinate (SFC) frame that is based on the mean IMF direction, mean solar wind velocity and bow shock model. The detailed definition of this coordinate system can be found in (Andres et al., 2015) and we thus limit ourselves to a brief description. The observations are rotated around the *X* axis to the plane containing the IMF vector and the IMF line tangent to the model bow shock surface and the point of tangency are found. The SFC coordinates of the spacecraft are: the distance of a spacecraft from the tangent line along the *X* direction and the separation of the intersection of the tangent line and the line going through the spacecraft location in the X direction from the point of tangency.

Meziane and d’Uston (1998) adopt the SFC frame and, assuming that the bow shock shape is best represented by a hyperboloid, performed a statistical investigation of the presence of reflected ions upstream the bow shock. They confirm that the intermediate ion foreshock boundary coincides with the ULF foreshock boundary reported by Greenstadt and Baum (1986) for the magnetic field cone angles (the angle between the IMF and velocity vectors) near $45^{◦}$.

Based on ISEE observations, Le and Russell (1992) suggest that the ULF foreshock boundary can be approximated by a straight line in the plane containing velocity and magnetic field vectors. This line starts at the point on the bow shock where the $\theta_{Bn}$ angle (the angle between the IMF direction and normal to the bow shock) is 50° and continues upstream with the slope that depends on the IMF cone angle. The directional angle of the boundary was not determined but theoretical considerations of Skadron et al. (1988) lead to the angle between this boundary and the Sun‐Earth axis of 78° for a Parker IMF orientation and such a value is visually consistent with Figure 10 in Le and Russell (1992).

Andres et al. (2015) used the Cluster magnetometer for statistical investigations of the ULF wave boundary and its dependence on the IMF cone angle. They use also the aforementioned SFC coordinate system but the principal difference is that they use the Farris et al. (1991) model for a determination of the bow shock location and shape. The authors specified the position of the ULF wave boundary in the SFC system and fitted it by a straight line in a short range of IMF cone angles. Their foreshock boundary is nearly perpendicular to the Sun‐Earth (the angle of $78^{◦}$ for the IMF orientation along the Parker spiral) and the results are generally consistent with those of Greenstadt and Baum (1986) and Meziane and d’Uston (1998).

Rojas‐Castillo et al. (2013) refer a foreshock compressional boundary associated with a strong compression of the magnetic field magnitude and density followed by significant decreases of these two quantities. In some cases, this boundary can coincide with the ULF wave boundary. However, the boundary does not always separate the unperturbed solar wind from a foreshock but rather marks the difference between large compressive ULF wave regions and the regions where the solar wind is slightly perturbed by small‐amplitude fluctuations.

Shan et al. (2018) investigate the existence of the foreshock boundary of quasi‐monochromatic ULF waves upstream of the Venus bow shock. To determine a spatial location of the ULF boundary, they transform the data from the aberrated solar ecliptic system into the SFC coordinate system introduced in terrestrial studies. Their results reveal a presence of the well defined boundary dividing the unperturbed solar wind upstream from the space occupied by ULF waves downstream this boundary. The location of the boundary is sensitive to the IMF cone angle and intersects the bow shock at quasi‐perpendicular geometries.

However, the SFC coordinate system is based on an estimation of the tangent line and tangent point to the bow shock model surface under a particular IMF direction. This definition excludes an application of hyperbolic models for some ranges of IMF cone angles. If the angle between the IMF vector and hyperboloid axis is lower than the flaring angle of the hyperbola the tangent point does not exist. For parabolic models, like Jelínek et al. (2012), the tangent point moves to infinity for the radial IMF and, consequently, a determination of its coordinates suffers with the large errors for small cone angles and the same is true for any other realistic bow shock model. On the other hand, the bow shock is perpendicular at the point of tangency but the foreshock formation requires a quasiparallel geometry. Urbář et al. (2019) employ another foreshock coordinate system, wherein one coordinate is represented by $\theta_{Bn}$ at the location where the IMF line coming through the spacecraft intersects the bow shock model surface. The distance of the spacecraft from the bow shock measured along this line serves as the second coordinate. The advantages of this system lie in the ease of localization of the point of intersection. Additionally, the error in a determination of this point is minimal, particularly for small cone angles. The fact that these coordinates cannot be applied on the observations of spacecraft that is not magnetically connected to the bow shock is not important for foreshock studies because the foreshock is formed at the magnetic field lines that intersect the bow shock.

Motivated by the advantages of the Urbář et al. (2019) coordinate system for foreshock investigations, we use it in the study of the location, shape and variations of the foreshock ULF wave boundary. We observe that the enhanced foreshock fluctuations occupy a defined space in these coordinates. The ULF boundary in this coordinate system also limits observations of enhanced fluxes of energetic ions. A transformation of the ULF boundary into geocentric coordinates provides a realistic shape of this boundary that is consistent with the idea of the foreshock formed by reflected and accelerated ions moving upstream along IMF lines that are advected antisunward with the solar wind flow. Finally, we discuss the dependence of the ULF boundary location on the upstream solar wind velocity and Alfvénic Mach number.

## 2 Data Selection and Their Processing

In the analysis, we apply observations of the THEMIS B and C spacecraft (THB and THC hereafter) during the 2007–2009 years when these spacecraft scanned appropriate distances upstream the dayside bow shock. The three‐axis fluxgate magnetometer provides the magnetic field vector with sampling rate up to 64 Hz (Auster et al., 2008) and the ESA instrument is designed to detect the ion velocity distribution in the energy range from 1.6 eV to 25 keV (McFadden et al., 2008) with a spin (3 s) time resolution. For an indication of energetic particle fluxes we checked also data of the SST telescope but we found that they are too noisy in some foreshock events. On the other hand, 15−25 keV protons measured by the two highest ESA energy channels cannot be mixed with the heavier ions for any reasonable solar wind velocity.

THEMIS observations were divided into 10‐min intervals and, since the spacecraft visit different regions, we compare magnetic field and plasma parameters with the solar wind values from the OMNI database. We select the intervals in which the mean magnetic field and plasma parameters differ by less than 15% from upstream values. We check that these margins are sufficient to exclude the magnetosheath and magnetosphere on one side, and to capture a potential modification of the plasma parameters in the foreshock (Urbář et al., 2019; Xirogiannopoulou et al., 2024) on the other side. Altogether, we collected more than 16,000 foreshock intervals.

As noted, our analysis is based on standard deviations of the IMF magnitude and its components computed over 10‐min intervals. The time resolution of plasma data is about 3 s, the magnetic field was averaged to the same resolution and thus the study covers the frequencies between 0.015 and 0.3 Hz that correspond well to the ULF range. Each interval is represented by one line in the database. This line contains information about the time, spacecraft location in GSE coordinates, the level of magnetic field fluctuations characterized by the standard deviation of the Alfvénic‐like components, $\sigma B={\left({\left(\sigma B_{x}\right)}^{2}+{\left(\sigma B_{y}\right)}^{2}+{\left(\sigma B_{z}\right)}^{2}\right)}^{1/2}$ where $B_{x}$, $B_{y}$, and $B_{z}$ are particular components, and their compressive component by the standard deviation of the magnetic field strength, $|B|={\left(B_{x}^{2}+B_{y}^{2}+B_{z}^{2}\right)}^{1/2}$, average dynamic pressure, $P_{d}$, average IMF vector, the $\theta_{Bn}$ angle computed at the intersection of the IMF line with the model bow shock surface (Jeřáb et al., 2005) and the distance of a particular observation from the model bow shock along the average IMF direction, $D_{BS}$. Since the mean magnetic field varies by an order of magnitude from event to event, we apply the approach of Šafránková et al. (2023) and use the standard deviations normalized to the mean magnetic field strength (σB/|B| and σ|B|/|B|) as a measure of the fluctuation amplitudes. Note that we do not use the bolded letters for vector quantities in the further text.

**Figure 1:**
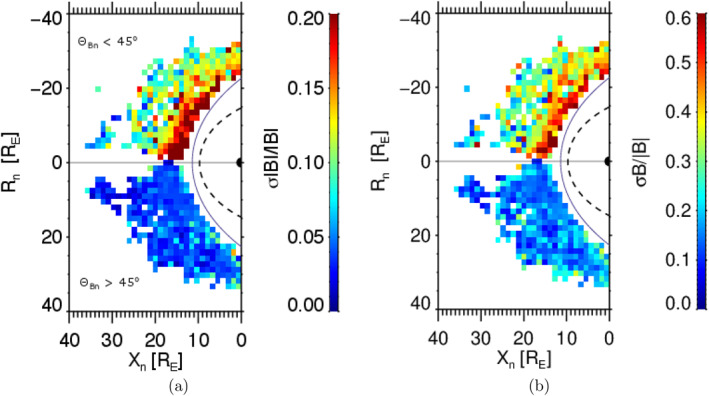
Spatial distribution of the normalized amplitudes of both compressive (a) and Alfvén‐like (b) magnetic field components in the $X_{n}-R_{n}$ plane. The solid line shows the model bow shock location calculated for the dynamic pressure of 1 nPa and the dashed black line marks the magnetopause position (see text for a description of the coordinate system).

The foreshock location depends on the bow shock position that is controlled mainly by the upstream dynamic pressure, $P_{d}$ whereas the IMF magnitude and Mach number, $M_{A}$ exhibit a minor effect under typical solar wind conditions (Jeřáb et al., 2005). In order to demonstrate a real spatial distribution of different parameters with respect to the bow shock, we expect the rotational symmetry around the $X_{GSE}$ axis and rotate all spacecraft positions into the X‐R plane, where $R={\left(Y^{2}+Z^{2}\right)}^{1/2}$. Since the foreshock fluctuations are expected upstream the quasi‐parallel bow shock, the sign of R is set according to the $\theta_{Bn}$ angle being positive for $\theta_{Bn}>45$° and negative for $\theta_{Bn}<45$°. To reflect the bow shock compression by the upstream dynamic pressure, $P_{d}$, the coordinates X and R are multiplied by ${P_{d}}^{1/6}$ and denoted as $X_{n}$ and $R_{n}$ in Figure 1. This “normalization” ensures that the model bow shock location would not change with the dynamic pressure in the $X_{n}-R_{n}$ coordinate plane.

The plots show the spatial distribution of the normalized amplitudes of fluctuations in the $X_{n}-R_{n}$ plane. The color scale represents the median fluctuation level in spatial bins (left—compressive and right—Alfvén‐like components). As it can be expected, the fluctuation levels of both components are low at the quasi‐perpendicular region (positive $R_{n}$) and roughly correspond to values found in the undisturbed solar wind (Šafránková et al., 2023), moreover, no change with the distance from the bow shock is observed. On the other hand, the fluctuation amplitudes of both components are enhanced by a factor of 2–4 in the whole quasiparallel region (negative $R_{n}$) and exhibit a clear enhancement toward the bow shock. The compressive fluctuations (Figure 1a) are typically three times lower than the Alfvén‐like (Figure 1b) ones (note that the color scales in right and left panels differ by a factor of 3) and it is true for the solar wind upstream quasi‐perpendicular bow shock as well as in the foreshock. Similar ratios were found by Narita et al. (2006) in the foreshock and by Šafránková et al. (2023) in the solar wind.

### 2.1 Foreshock Coordinate System

**Figure 2:**
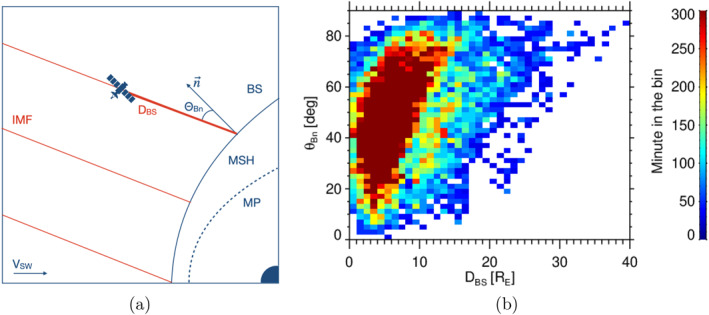
Estimations of foreshock coordinates (a) and data coverage in the new foreshock coordinates (b).

Figure 1 combines in each bin the intervals with different $\theta_{Bn}$ but the foreshock geometry would strongly depend on this angle. For this reason, we use the foreshock coordinate system based on $\theta_{Bn}$ and a distance from the spacecraft to the model bow shock along the IMF direction, $D_{BS}$. The definition of the coordinates is sketched in Figure 2a. It should be stressed out that our coordinates cannot be determined for the points that are not magnetically connected to the bow shock and it reduces the number of intervals for the statistics. Nevertheless, as Figure 2b demonstrates, we still have hundreds of minutes in the region of interest and the only problem is the coverage of very low $\theta_{Bn}$ angles.

### 2.2 Determination of Foreshock ULF Boundary

**Figure 3:**
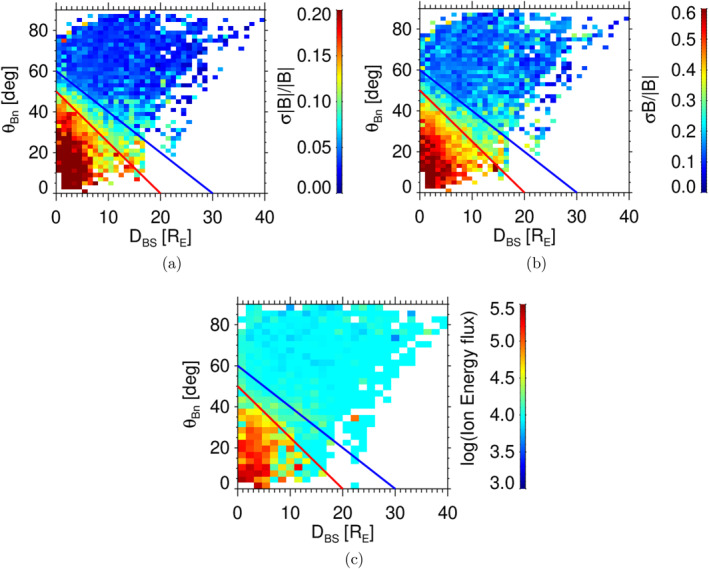
Distributions of the average fluctuation amplitudes (color scales) in the $\theta_{Bn}-D_{BS}$ coordinates: (a)—σ|B|/|B|, and (b)—σB/|B|. The panel (c) shows the distribution of the energy flux of energetic ions in the same coordinate system. The colored lines stand for the strong (red) and weak (blue) foreshock boundaries.

Figure 3 presents distributions of the normalized amplitudes of compressive (Figure 3a) and Alfvén‐like (Figure 3b) fluctuation components in the $\theta_{Bn}-D_{BS}$ coordinates; the color scale represents the median values in coordinate bins; we note that the bins containing less than three intervals are marked by the white color. The normalized fluctuation amplitudes of both components for a given $\theta_{Bn}$ angle peak at the bow shock and decrease with the distance from the bow shock. If $D_{BS}$ is kept constant, the decrease of the fluctuation level with $\theta_{Bn}$ is evident in both panels. The color scale suggests that the transition from a region occupied by enhanced fluctuations to the region resembling the solar wind fluctuation level is gradual and thus it is a question where the ULF boundary would be put. For this reason, we define two boundaries—strong and weak. Due to the spread of the data, we decided to fit the position of the boundary with the polynomial fit of the first order and to fix the $\theta_{Bn}$ value at $D_{BS}=0$ to 50° for our strong foreshock boundary, in agreement with Le and Russell (1992). Then, we follow the Šafránková et al. (2023) analysis of the fluctuation amplitude, apply the median level of normalized solar wind fluctuations (0.05 for compressive and 0.15 for Alfvén‐like fluctuations) and determine a position of the boundary as the location where the median fluctuations are by a factor of 1.5 above this level for weak and 3 for the strong boundary. Since the coverage of the data is very far from a uniform case, we weighted each point with the observation time in the bin. It actually means that only the points for $D_{BS}<10$ are decisive for the boundary determination (see Figure 2) and therefore we rounded the result to the nearest tenth. Figure 3 suggests that a slightly enhanced level of fluctuations can be observed also above our foreshock boundaries but we use 10‐min averages of the magnetic field measured in the foreshock, thus the majority of events exhibiting enhanced fluctuations at $\theta_{Bn}>50$° are probably related to uncertainty in a determination of this angle. On the other hand, the fluctuations are low and their amplitude is not influenced by foreshock processes above the blue line; this area can be thus attributed to non‐modified solar wind and used for a comparison albeit these points are still magnetically connected to the bow shock. As we already noted, the Alfvén‐like fluctuations are larger than the corresponding compressive components but their distributions in the foreshock coordinates are similar. The distribution of energy fluxes of the ions with energies of 15−20 keV registered by the ESA instrument shown in Figure 3c confirms the fact that the ion foreshock boundary roughly coincides with our strong ULF boundary.

**Figure 4:**
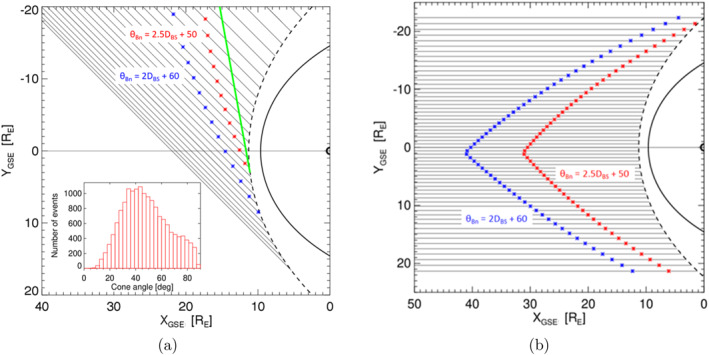
Sketch of the foreshock Ultra‐low frequency boundary under typical solar wind conditions for IMF oriented along the Parker spiral (a) and for radial IMF (b). The insert in panel 4a implies the distribution of the cone angles in our data set.

Although the presence of a foreshock boundary is clear in Figure 3, it is difficult to image where the boundary lies in the space. It is evident that its position depends on the bow shock location (i.e., on factors influencing its dimensions and shape) and on the IMF cone angle. For this reason, Figure 4a shows the location of the model boundaries: bow shock (Jeřáb et al., 2005)—dashed line, magnetopause (Shue et al., 1997)—full line for typical conditions defined as it follows: the solar wind dynamic pressure, $P_{d}=1$ nPa, Alfvénic Mach number, $M_{A}=8$, |B|=5 nT, $B_{z}=0$ and IMF cone angle = 45°. The distribution of IMF cone angles in the insert shows that it corresponds approximately to a mean value in our set. The colored dots signify the positions of foreshock ULF boundaries (strong foreshock: red points and weak foreshock: blue points) as defined in Figure 3. The green line marks the position of the ULF boundary suggested by Skadron et al. (1988) and we can note a good matching with our strong ULF foreshock boundary. As already noted, the boundary determined by Andres et al. (2015) would represent approximately a vertical line in our plot and its position would change with the IMF cone angle.

The dependence of the foreshock ULF boundary on the cone angle demonstrates Figure 4b showing its shape for the cone angle equal to zero. Note that the used bow shock model (Jeřáb et al., 2005) is not axisymmetric in GSE coordinates and it is reflected also in the dawn‐dusk asymmetry of the boundary. It should be stressed out that this plot serves only for a very approximate idea how the boundary changes with the cone angle. Our set does not contain enough data for very small cone angles and larger distances from the bow shock and thus we cannot exclude that the foreshock extends farther upstream under these conditions as simulation results suggest (Dorfman et al., 2023; Palmroth et al., 2015).

## 3 Variations of the ULF Boundary: Statistical Analysis

### 3.1 Foreshock Boundary and Upstream Velocity

**Figure 5:**
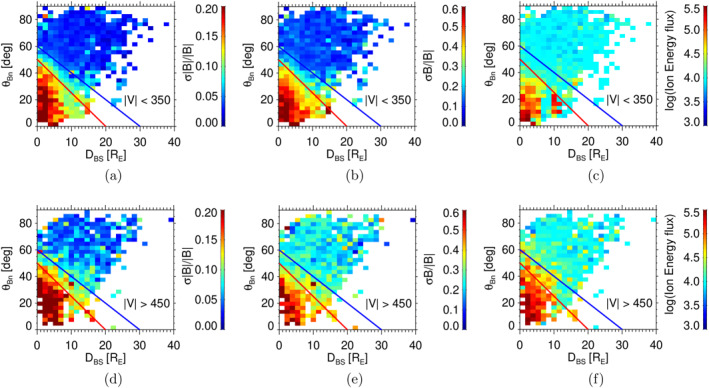
Normalized Ultra‐low frequency wave amplitude and the energy flux of energetic particles in the foreshock coordinates for low (<350 km/s, top panels) and high (>450 km/s, bottom panels) upstream solar wind velocities. The format of the figure follows Figure 3; the foreshock boundaries are taken from Figure 3 to guide the eye in comparisons.

The shape of the boundaries well corresponds to the expected mechanisms leading to their creation that can be briefly summarized as it follows. The particles reflected at and energized by the bow shock move upstream along the magnetic field lines. These lines are advected downstream by the solar wind flow and it leads to boundary curvature. However, its position would depend on the upstream velocity that pushes the field line closer to the bow shock for larger solar wind velocities and on the parallel velocities of the reflected particles because larger parallel velocities would result in a less curved boundary located farther from the bow shock. On the other hand, the guiding center velocity of reflected particles is roughly proportional to the solar wind velocity but the proportionality constant is influenced by the $\theta_{Bn}$ angle (Andres et al., 2015). Since it is not clear which of these two effects prevails, Figure 5 presents two subsets for low (<350 km/s, top panels) and high (>450 km/s, bottom panels) upstream velocities. The format of the panels including positions of foreshock boundaries follows Figure 3. However, we should note the presence of high fluctuations above the strong foreshock boundary for larger velocities (bottom panels) whereas the low fluctuations often occupy the space below this boundary for a subset of lower velocities (top panels). It is observable better for compressive fluctuations (left panels) because the typical level of Alfvén‐like fluctuations in the solar wind strongly increases with the velocity.

A brief survey of Wind observations at the L1 point reveals that the mean σB/|B| in the slow wind is about 0.1 but it increases to 0.2 in the fast wind and these numbers are fully consistent with our observations upstream quasi‐perpendicular bow shock. This effect partly masks the velocity influence on the shift of the foreshock boundary because we are keeping the color scales. Similar patterns can be found in the plots of energetic particles (Figures 5c and 5f). Nevertheless, we can conclude that the foreshock boundaries move slightly inward if the velocity decreases. It suggests that the proportionality constant between the solar wind velocity and the velocity of reflected ions is larger than unity, in agreement with the analyses of Bonifazi and Moreno (1981) and Andres et al. (2015).

### 3.2 Mach Number Dependence

**Figure 6:**
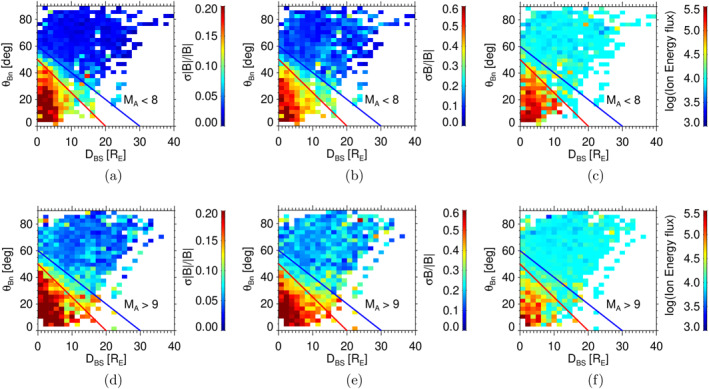
Normalized Ultra‐low frequency wave amplitude and the energy flux of energetic particles in the foreshock coordinates for low (upper panels) and high (lower panels) $M_{A}$. The format of the figure follows Figure 3; the foreshock boundaries are taken from Figure 3 to guide the eye in comparisons.

As reflection of particles from the bow shock is affected by the Alfvénic Mach number, $M_{A}$, we created also two subsets—for $M_{A}<8$ (top panels) and $M_{A}>9$ (bottom panels) and show the distributions of the fluctuation amplitudes and energetic particles in Figure 6. The figure uses the same format as Figure 5 but the data are sorted according to $M_{A}$. One can note that the decrease of $M_{A}$ results in the shift of the ULF foreshock boundary toward the bow shock. It seems reasonable because high‐$M_{A}$ shock would be more effective in reflection and acceleration of the particles. However, the distributions of energetic particles shown in Figures 6c and 6f reveal that the larger flux of energetic particles in the studied energy range is observed in a low‐$M_{A}$ foreshock.

### 3.3 Evaluation of the Velocity and Mach Number Effects

**Figure 7:**
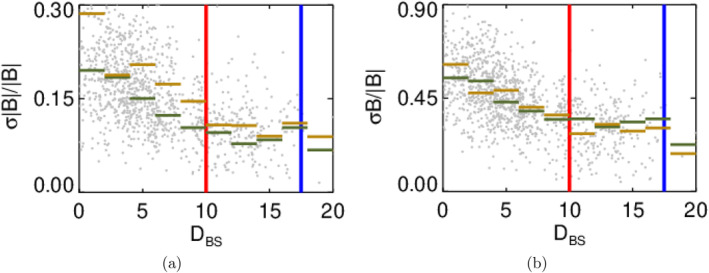
Amplitudes of compressive (left) and Alfvén‐like (right) fluctuations as a function of distance from the bow shock in two velocity bins: V<350 km/s (green bars) and V>450 km/s (yellow bars) in the $\theta_{Bn}$ range of 20°−30° of $\theta_{Bn}$. The foreshock boundaries from Figure 3 are shown by the vertical lines with corresponding colors.

Figure 5 indicates that both foreshock boundaries move outward if the solar wind speed increases and this effect was attributed to an increasing energy of particles reflected from the bow shock that are responsible for excitation of foreshock waves. Since it is difficult to quantify changes shown as colors in Figures 5 and 7 presents the variation of the fluctuation level with $D_{BS}$ for compressive (7a) and Alfvén‐like (7b) fluctuations in the narrow range of $\theta_{Bn}$ between 20° and 30°. The vertical red and blue lines represent the locations of the corresponding foreshock boundaries for $\theta_{Bn}=25$° and colored bars stand for the median level of fluctuations in the $D_{BS}$ bins for subsets of low (<350 km/s, green) and high (>450 km/s, yellow) upstream velocities. The panels show that whereas the upstream velocity strongly increases the level of compressive fluctuations at the bow shock (the ratio of medians for subsets of high and low velocity is 1.5), the velocity influence becomes negligible behind the strong foreshock boundary. It is interesting to note that the Alfvén‐like fluctuations are affected by the velocity only weakly through the whole foreshock.

**Figure 8:**
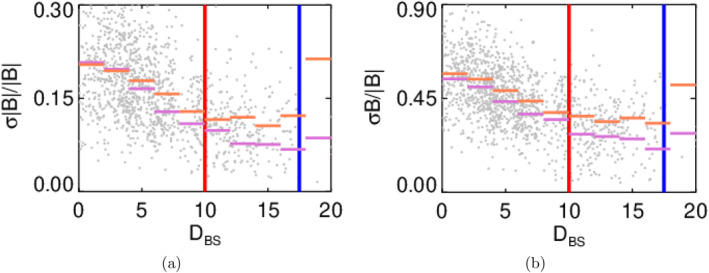
Amplitudes of compressive (a) and Alfvén‐like (b) fluctuations as a function of distance from the bow shock for two $M_{A}$ ranges: $M_{A}<8$ (violet bars) and $M_{A}>9$ (orange bars). The foreshock boundaries from Figure 3 are shown by the vertical lines in corresponding colors; the gray dots belong to individual intervals and demonstrate the spread of fluctuation amplitudes.

A similar analysis of the $M_{A}$ effect on the location of a foreshock boundary shown in Figure 8 brings unexpected results. The figure shows the dependence of the fluctuation level on upstream $M_{A}$ (note that the format of the figure follows Figure 7, violet bars stand for a subset of $M_{A}<8$ and orange ones for $M_{A}>9$). It is surprising that whatever larger $M_{A}$ results in larger fluctuation levels of both compressive and Alfvén‐like components throughout the whole foreshock (Figure 6), this effect is weaker at the bow shock and enhances with $D_{BS}$. The median fluctuation levels for these two subsets differ by a factor of 1.8 at the weak foreshock boundary. It would be noted that also the level of energetic particle fluxes is lower for a subset of events with $M_{A}>9$ (Figure 6).

## 4 Discussion

Our study deals with the position of the foreshock ULF wave boundary and with factors that can affect this position. Figure 1 presents our data set and demonstrates that the enhanced ULF fluctuations are observed in the hemisphere where $\theta_{Bn}<45$° and their amplitude increases toward BS. It was shown, for example, by Andres et al. (2015) (and references therein), that the location of the boundary depends on the IMF cone angle and thus it cannot be identified in the plot that combines all cone angles. Although we use all relevant THEMIS observations, the set is not large enough to be divided to subsets for the narrow ranges of cone angles and to demonstrate the location of ULF boundary by this way. In order to detect the ULF wave boundary location, we apply the foreshock coordinates suggested by Urbář et al. (2019) and plot the amplitude of fluctuations in the $\theta_{Bn}-D_{BS}$ space in Figure 3. The plots show that the median amplitudes of compressive as well as Alfvén‐like fluctuations in the coordinate bins are well organized and that the used color scale allows us an identification of the ULF foreshock wave boundary. Since the boundary is not sharp, we define the strong foreshock as a region where the amplitude of a compressive component of foreshock magnetic field fluctuations (quantified as σ|B|/|B|) exceeds 0.15 and the weak foreshock as the space where this amplitude lies in the range of 0.075<σ|B|/|B|<0.15. Such boundaries can be approximated by straight lines that are shown in Figure 3a by red and blue color lines. The other two panels demonstrate that the same boundaries can be applied for the Alfvén‐like fluctuation component and for the energy flux of particles in the range of 15−20 keV. The position and shape of the boundaries in the GSE coordinates are shown in Figure 4 for two IMF cone angles (Parker spiral and radial IMF orientations) and compared to the already published results (Andres et al., 2015; Greenstadt & Baum, 1986; Le & Russell, 1992; Skadron et al., 1988).

The definition of two foreshock boundaries apparently contradicts to the aforementioned studies that reported a distinct sharp transition from the undisturbed solar wind to foreshock. These studies were based on the identification of the transition from the solar wind to foreshock in the time series but our definition is based on the statistical processing of the median values of standard deviations computed over 10‐min intervals. Due to large variations of the magnetic field direction, the $\theta_{Bn}$ angle as well as $D_{BS}$ change in broad ranges within this interval (Meziane et al., 2004) but their medians are used for further processing. A visual check of several intervals in the space between our two boundaries suggests that it is mostly populated by events where the foreshock fluctuations occupy only a part of our 10‐min interval, probably due to changes of the magnetic field direction. Other sources of uncertainties in the $\theta_{Bn}$ determination are variations of the normal direction connected with the local bow shock deformations caused by strong fluctuations of the ion density (or velocity) (Němeček et al., 2023). It means that our weak foreshock boundary would be rather interpreted as a most distant point where the foreshock fluctuations can be temporarily found under given averaged upstream conditions.

**Figure 9:**
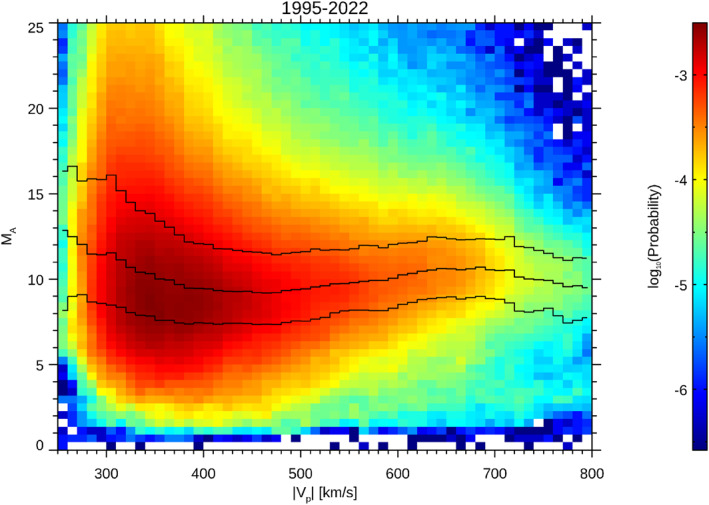
A 2D histogram of the occurrence rate (color scale) of a combination of the solar wind bulk speed, $\left|V_{p}\right|$ and corresponding $M_{A}$. The black bars show median values in the speed bins and their first and third quartiles.

Our study of the solar wind velocity and Mach number effects seems to contradict intuitive expectation that the high values of Mach number would be associated with fast solar wind streams. Since the solar wind speed and $M_{A}$ are not fully independent quantities, Figure 9 presents the 2D histogram of occurrence rates of combinations of $M_{A}$ and solar wind speed. This histogram uses about the 20 years of continuous solar wind observations by the Wind spacecraft; the occurrence rate is shown by the color scale and the medians in the speed bins by black bars. One would intuitively expect that the occurrence rate of high‐$M_{A}$ events would increase with the speed but the figure shows an opposite trend and $M_{A}$ exceeding 12 are observed nearly exceptionally in the slow wind. It is reflected also by larger median values of $M_{A}$ for lower speeds.

The prevalence of the occurrence rate of high‐$M_{A}$ events at low velocities can explain a negligible increase of the fluctuation level at the bow shock for these events, but the level of fluctuations for $D_{BS}>18\;R_{E}$ (Figure 8) requires further investigations because Figure 6 indicates very low fluxes of energetic particles in our energy range (15–20 keV).

## 5 Conclusion

The statistical study of a location of the ULF foreshock boundary and the behavior of compressive and Alfvén–like components of the magnetic field fluctuations in the foreshock can be summarized as it follows:

1. The ULF foreshock boundary is well defined in the $\theta_{Bn}-D_{BS}$ coordinate system.
2. It tends to shift outward with an increasing solar wind bulk speed.
3. The increasing solar wind speed enhances the fluctuation level mainly at a close proximity of the bow shock and this effect is more noticeable for the compressive fluctuation component.
4. The boundary also moves outward with increasing $M_{A}$.
5. The increasing $M_{A}$ leads to enhancement of the fluctuation level at the foreshock boundary but the $M_{A}$ influence is negligible at the bow shock.

It should be noted that the used data set does not contain enough data gathered under very low $\theta_{Bn}$ angles, thus the above conclusions should be applied with a care on the foreshock boundary under the radial IMF orientation.

## Data Availability

The magnetic field and plasma data are public and are available online via http://cdaweb.gsfc.nasa.gov/; details: THEMIS‐ARTEMIS data at cdaweb. gsfc.nasa.gov/pub/data/themis; and the Wind data can be accessed via cdaweb. gsfc.nasa.gov/pub/data/wind, all using the particular instruments and time intervals of events for an identification.
